# New trends in science communication fostering evidence-informed policymaking

**DOI:** 10.12688/openreseurope.14769.2

**Published:** 2023-10-24

**Authors:** Gábor Szüdi, Pamela Bartar, Gorazd Weiss, Giuseppe Pellegrini, Marina Tulin, Tessa Oomen

**Affiliations:** 1 Zentrum für Soziale Innovation, Wien, 1150, Austria; 2 Observa - Science in Society, Vicenza, 36100, Italy; 3 Communication Science Department, University of Amsterdam, Amsterdam, 1018, The Netherlands; 4 Department of Media and Communication, Erasmus University Rotterdam, Rotterdam, 3062, The Netherlands

**Keywords:** science communication, evidence-informed policy-making, trust, dialogue model, open science, visualisation, digitalisation

## Abstract

This article aims to assess novel trends in science communication relating to how policymakers in the field of innovation and digitalisation policy consume and use scientific findings. We investigate the mutual influence that science communication and policy have on each other and answer the question how emerging science communication trends in the science-policy nexus might influence the use of scientific findings in the policymaking process.

By using Google and the largest scholarly repositories, Google Scholar, ResearchGate, and Academia.edu, from 1 ^st^ March to 31 ^st^ May 2020, we reviewed policy documents and academic literature containing relevant information on the evolution of characteristics of global, European, and national science communication activities and the interrelated policy responses to identify the most relevant current trends in the evidence-to-policy process alongside three key challenges; trust, translation, and timing. The three identified main trends are (1) a stronger engagement between science and policy, (2) more open, reliable, and accountable science communication practices with policymakers, and (3) the increasing digitalisation and visualisation of science communication.

We deepened our investigation by conducting online semi-structured interviews with relevant policy stakeholders at the international and national level between 1 ^st^ May and 31 ^st^ July 2020. With the support of the European Commission and building on the existing network of partners, we identified decision-makers and advisors with relevant experience in fields related to innovation and digitalisation policy working in four countries that represent different levels of generalized social trust: Austria, Italy, Hungary, and the Netherlands, and at the international/European Union level.

After an online consultation process with a global set of policymakers, these theoretical findings were translated to policy recommendations showcasing possible solutions in science communication that may be initiated, strengthened, or continued by policy stakeholders in order to reach a more effective and efficient uptake of scientific findings in evidence-informed policymaking.

## Plain language summary

TRESCA – Trustworthy, Reliable and Engaging Scientific Communication Approaches – is a research project aimed at understanding how science communication can help re-build trust in science and scientists. The project wants to create positive changes through common research activities with various stakeholders, e.g., the general public, scientists, journalists, and policymakers.

Thus, TRESCA also aimed to identify the most important actual trends how communication between scientific experts and policymakers changed in the last decades in the field of innovation and digitalisation policy. We looked at how these trends might influence the way policymakers receive, interpret, and use scientific evidence during their daily work.

The partners first checked various scientific and non-scientific documents concerning potential new communication trends between scientists and policymakers. The partners conducted interviews with policymakers working in four European countries (Austria, Hungary, Italy, the Netherlands) and at the international/EU level. The interviews investigated the scientific data sources, data collection processes, science communication topics, channels, and formats frequently used by policymakers.

We found that at least three new trends had strengthened in the last decades: (1) increasingly often more permanent formal relationships are developed between scientists and policymakers to cope with the more frequent and intense communication; (2) to enhance trust between scientists and policymakers, more transparent and reliable communication channels and formats are used; (3) policymakers need to understand more scientific information in less time therefore visual and digital communication formats are getting more widespread.

After an online consultation process, practical recommendations were provided to policymakers on how to support more effective communication with scientists. This included the creation of more training opportunities, the increased use of communication guides, the promotion of fact-checking websites, or ways to motivate scientists to communicate with policymakers. These steps might support a novel communication process built on trust and the understanding of each other’s perspective.

## Introduction

The aim of this article is to assess the ongoing and emerging communication trends between scientists and policymakers in the field of innovation policy and digitalisation from the perspective of evidence-informed policymaking. The production of scientific evidence and the policy-making process could mutually influence each other and we describe the most relevant current science communication trends coming into being due to this mutual relationship.

During the pandemic we have witnessed an intense relationship between decision makers and scientists. As the scientific journal Nature ( Howe, 2020) has pointed out several times, politics and science are inseparable even if they have different objectives. Never as in this period, therefore, is it of vital importance to understand more deeply the processes of relationship and development between these two actors whose work is more and more intertwined in the public scene. The starting point of our trend analysis is that scientific evidence is one key element in the policy-making process, but under no circumstances the only one ( Gluckman, 2018; Pielke, 2007). Policymakers operate under definite organisational circumstances and mandates specific to each country and each policy field, possess relevant (policy and scientific) experience, as well as cultural and social predispositions, values, and beliefs that may all influence their *use* of scientific information in the policy-making process ( Gluckman, 2018).

When focusing on the ‘use’ of scientific evidence while discussing the communication trends in the science-policy nexus, we follow the policy argument stating that – since it is in most cases too complicated to ascertain how much of a role certain scientific inputs played in a specific policy decision – it is sufficient to assess whether science communication reached its basic objective, i.e., that relevant data or information has been received and understood by those institutions and persons making policy decisions ( National Research Council, 2012). We therefore apply this ‘use’ approach in this article and – in line with Weiss *et al.* (2005) – consider whether and how the scientific information was received and processed in a formal policy-making process.

Due to these two analytical considerations, i.e., acknowledging that scientific evidence is just one element for policymakers in their policy-making process, and putting the focus on the understanding and processing of scientific input by policymakers, we use the term ‘evidence-informed’ policy-making – in place of the more widespread term ‘evidence-based’ policymaking when discussing the scientific communication with policymakers in this article. Policy decisions are informed by scientific evidence but are not necessarily based on them.

The actual impact of scientific evidence in policy-making processes is highly dependent on the way the information is communicated and presented to policymakers. No matter how robust and convincing scientific evidence may be, issues related to collaboration, relationships, access to the good and timely information, and planning need to be considered and addressed for a successful understanding and uptake of scientific input by policymakers. Following the framework developed by Hinrichs-Krapels *et al.* (2020), we grouped the issues to consider for an improved evidence-to-policy process into three principles, i.e., trust, translation, and timing.

Since scientists and policymakers mostly do not work together on a continuous basis, building trust and understanding between stakeholders is crucial. Trust can initially be based on the mere reputation of respected scientific organisations or individuals, or developed with the support of intermediary organisations, but in the longer term it should be enhanced by establishing collaborations and good relationships between scientists and policymakers.

As all individuals, policymakers are influenced by their experience, values, beliefs, cultural, and social predispositions which are all key factors when seeking the use of scientific evidence ( Davies, 2015). This is coupled with the institutional settings with its own logic, rules, and conditions where policymakers make their decisions, adding another layer to the complexity of evidence-informed policymaking. These individual and institutional characteristics may result in situations where policymakers have access to and understand all available scientific information but they still do not use (or underuse) science in delivering policy options ( National Research Council, 2012).

Henry (2011) shows that this may happen when “biased assimilation” develops within a network of policymakers with similar experiences and insights (usually in a specific policy field). Policymakers tend to interpret scientific evidence in a way that supports their prior beliefs and values, which is also called confirmation bias or motivated reasoning ( Schlosser *et al.,* 2021).

Such network-level biased assimilation is influenced more by the ‘institutional’ values, preferences and strategies formed within formal structures and networks than by the policy-makers’ ‘individual’ beliefs and perception. Scientific input is filtered through these shared values and preferred strategies, which makes it difficult for new scientific evidence to change the established interpretation of policy-relevant information ( Henry, 2011).

Trust-building between scientists and policymakers enable us to break down such ‘biased assimilation’ if sufficient conditions are met. Henry & Dietz (2011) considers that a minimum set of shared key values and beliefs between scientists and policymakers are a necessary, but not a sufficient, pre-condition for trust-building but in addition, policymakers should also associate relevance and influence with a specific scientific field, institution, or stakeholder to deem scientific input more trustworthy. In line with these findings, a recent Joint Research Centre (JRC) report highlighted these complex values and conditions which permeate the context of policymaking processes and stressed the need for scientists to be more aware of the way policymakers frame scientific evidence ( Mair *et al.,* 2019).

Thus, effective science communication practices must first check, assess, and understand the construction and aims of the underlying formal structure in which a policy decision is being made, rather than focusing on individual policymakers’ goals or characteristics. This structure does not only involve the organisation itself within which a policymaker operates, but also the network of organisations and individuals within which this organisation is embedded ( Hinrichs-Krapels *et al.,* 2020).

Understanding which types of beliefs and values are more or less prone to ‘biased assimilation’ and how the structure of policy networks may influence and is influenced by values and beliefs may help scientists to build trust with policymakers. The more complex the policymaking structure, the more difficult it might get for policy actors to methodologically assess the trustworthiness of new scientific evidence, which is principally due to the involvement of more stakeholders and hence more interests and strategies in the policy-making process ( Henry, 2011). 

It can be beneficial for trust-building when such organisations aim to arrange teams of relevant stakeholders of the science-policy nexus to learn from each other in order to better understand and utilize the value and impact of scientific knowledge in policy-making. Team members with different expertise, competencies, capacities and experiences can pull together and master relevant skills to support evidence-informed policy. Such skills might be manifold and interrelated; for instance, the Knowledge Management Policy (KMP) initiative of JRC identified eight such skills, ranging from research synthesis to interpersonal skills or effective communication of knowledge ( Topp *et al*., 2018).

The purpose of science communication within policymaking should go beyond the mere presentation of scientific knowledge. Translation of scientific input means the ability of scientists to translate academic research to understandable and relevant messages in an accessible and readable way ( Oliver & Cairney, 2019).

Scientists involved in communication with policymakers should consider several interrelated aspects to facilitate a proper translation ( Hinrichs-Krapels *et al.,* 2020). First, language is of utmost importance: scientific jargon should be toned down to the minimum and wording should be re-aligned with the interests and needs of the policy audience. Second, scientists should also understand what the compelling arguments are from the perspective of policymakers. If the aim is to enable the use of scientific results in the policy-making process, the communicators should strive to understand the interests and needs of the policymakers and the policymaking process itself (to break down biased assimilation). In other words, the research should be presented in a way that demonstrates its applicability to the specific circumstance at hand ( Moore *et al.,* 2009), also referred to as finding relevance for the evidence ( Cartwright & Hardy, 2012). An efficient trust-building process can greatly enhance the success of the translation process: policymakers will be more open about their actual agenda, and scientists will feel encouraged to be more explicit about their methodologies, limitations, and weaknesses ( Whitty, 2015).

While theoretically it may seem straightforward it is still often difficult for scientists to find the right language and format in which to communicate their evidence to policymakers. This happens because scientists have a distinctive way of interpreting, describing, and presenting evidence which is suited for peer communication but less so for outreach communication with policymakers or even with the general public ( Dudley *et al.,* 2021).

As the recent COVID-19 pandemic demonstrated, this already existing communication ‘gap’ may be further exacerbated by the rising level of available scientific information (‘infodemic’), which makes it near impossible for policymakers to consider all evidence relevant to decisions ( Williams *et al.,* 2020).

The actual use of the scientific findings is also influenced by timing. Policymakers often operate on a tight schedule, under pressure from their policy networks and the general public while they have to make sense of a growing amount of frequently inaccessible or contradicting evidence ( Hinrichs-Krapels *et al.,* 2020). The formal structures of policymaking are also getting more complicated, with several levels and types of government being involved in policymaking in fields such as innovation policy and digitalisation in Europe. In addition to trust-building and proper translation, the continuous challenge for scientists, as outlined by Davies (2015), is to “identify the best available evidence in the time frame in which decisions have to be taken, whilst also developing a more robust evidence base for future policy-making in the medium to longer term”.

Based on this theoretical framework, we pose the following research question in this article: How do emerging science communication trends in the science-policy nexus influence the use of scientific findings in the policy-making process for digitalization and innovation domains?

As mentioned, the goal of science communication with policymakers is to ensure the understanding, use, and uptake of scientific evidence in policy decisions, which is often aggravated by several circumstances. Our theoretical frame summarized these aggravating conditions in the categories of trust, translation and timing and – as we will see within the result section – the main identified trends of science communication towards policymakers seek to address one or more of these three issues.

We aimed to go beyond the current state-of-the-art research in this field by not only understanding the emerging science communication trends but also by contributing to a potentially more efficient use of scientific findings in evidence-informed policymaking, taking into account the results of our qualitative study. This was enabled by our continuous engagement of relevant policymakers in the research process, which supported to pinpoint the key challenges and the potential solutions to facilitate evidence-informed policymaking by better science communication.

## Methods

### Ethical approval

This study was approved by the Research Ethics Review Committee of the Erasmus School of History, Culture, and Communication. Written informed consent was obtained from all participants prior to the study.

### Study design

To address the above-mentioned research question, the partnership conducted a qualitative study by using a methodological triangulation process within the TRESCA (“Trustworthy, Reliable, Engaging Science Communication Approaches”) project funded under the Horizon 2020 framework programme. ^1^

First, comprehensive secondary research was conducted where relevant national and international-level documents – including peer-reviewed articles, studies, non-academic publications, white papers and project reports, or online sources such as websites and project repositories – were consulted from 1 March to 31 May 2020. Not limiting their search to academic literature, the partners aimed to collect these types of documents containing relevant information on the past and ongoing evolution of topics, channels and other characteristics of global, European and national science communication activities and the interrelated policy responses.

For non-academic papers, most importantly, a thorough web search with Google search engine was utilized, with project documents and related policy papers also checked through the Community Research and Development Information Service (CORDIS) ( https://cordis.europa.eu/), the biggest public repository of EU-funded research and innovation projects. Regarding academic papers, the most common scholarly repositories were visited, i.e., Google Scholar, ResearchGate, and Academia.edu. These web search engines were selected because they were deemed the most comprehensive in the topics based on the previous experiences of the partners. Involved researchers from the Erasmus University Rotterdam also used its own search engine for libraries called sEURch ( https://www.eur.nl/en/library/seurch).

The main inclusion criterion was that the documents should contain new insights and perspectives for our research question (“How do emerging science communication trends in the science-policy nexus influence the use of scientific findings in the policy-making process for digitalization and innovation domains?”). Specific focus was given to findings from social sciences and humanities (SSH) research concerning science communication developments in relation to innovation and digitalisation policy. The desk research was foreseen to be as broad as possible but the partners exchanged documents during the process and agreed to exclude documents that were not strictly related to innovation and digitalisation (too general), considered outdated, or biased. Such exclusions have always happened with mutual agreement.

In order to find relevant documents, key words and expressions in the topics of science communication, science-policy nexus, evidence-informed policymaking, and innovation and digitalisation were agreed upon by partners, and used to identify useful documents publicly available. The key expressions included public communication of science, science communication with policymakers (also in different variations such as communicating science to policymakers or scientific advice to policymaking), evidence-based policy, science-based policy, trust (in science and scientific experts), the role of science in decision-making, citizen engagement in science, open science, responsible research, and innovation. The partners held online meetings during the desk research process to discuss their findings and added new key expressions to their search when observing potential common trends among countries, for instance visualisation, digitalisation, or (fighting against) misinformation.

By consulting these documents, we aimed to identify the key emerging and ongoing science communication trends resulting from the mutually reinforcing relationship between science communication and policymaking in fields related to innovation policy and digitalisation (which belonged to the core topics of the TRESCA project). The focus lied on such trends that substantially changed – or have the potential to change – the relationship between policymakers and scientists through new or improved methods of communication, facilitating evidence-informed policymaking.

We focused on two main periods: recent and visible trends that have been shaping since the mid-1990s, and the trends emerging at the time of report writing (after the first waves of the COVID-19 pandemic). This distinction became necessary because of the outbreak of the pandemic which significantly distorted the topics, channels and other characteristics of international and national science communication efforts and the interrelated policy responses. The aim was to understand how communication in the science-policy nexus contributed to shaping the process of evidence-informed policymaking in recent decades, with particular attention to the ongoing COVID-19 pandemic. In this regard, the focus was on both visible trends shaping up since the mid-1990s, as well as on emerging trends at the time of compiling the data (mid-2020) – this distinction was necessitated by the disruptive effects of the first wave of the COVID-19 pandemic. The mid-1990s as starting point for the data gathering and assessment was specified because of its perceived importance by several experts as a trend shift when science communication began its currently ongoing transformation enabled by more advanced ICT and digital technologies fostering upscaling and dissemination ( Bucchi & Trench, 2015).

More than hundred documents selected with the help of such key expressions were checked, the most important of which are listed as references at the end of this article, while all documents are listed under Szüdi *et al.*, 2022c. These documents are publicly available for researchers interested in understanding the source of our results in more detail. When reaching the pre-determined project deadline for mapping and collecting the required documents (31 May 2020), each partner extracted the key findings of the analysed documents and summarized the identified trends in a project report ( Szüdi *et al.,* 2020). The trends identified through secondary research were in the second step reviewed, validated, and extended by semi-structured online interviews conducted with a broader defined group of policymakers dealing with innovation policy and digitalisation at the international and national level in four selected countries, i.e., Austria, Hungary, Italy and the Netherlands (see Table 1) from 1 May to 31 July 2020. The countries involved in the secondary research and interview process were selected in a way to satisfy several conditions. The countries represented a balanced geographical coverage (Western Europe, Southern Europe, Central and Eastern Europe), as well as a mix between countries with high (NL), medium (AT) and low social trust (IT, HU) based on data from the European Social Survey (ESS). ^2^

**Table 1.** Overview of interview partners by country, type, and organisational level.

No.	Country or international level	Type of policymaker *	Organisational level
1	Austria	Policymaker	National
2	Austria	Policymaker	National
3	Austria	Policymaker	National
4	Austria	Policymaker	Regional
5	Austria	Policy advisor	National
6	Austria	Knowledge coordinator	Municipal
7	European Union	Policymaker	European Commission
8	European Union	Policymaker	European Commission
9	European Union	Policymaker	European Commission
10	European Union	Policymaker	European Commission
11	European Union	Policy advisor	Independent EU body
12	European Union	Knowledge coordinator	EU delegation
13	Hungary	Policymaker	National
14	Hungary	Policymaker	National
15	Hungary	Policy advisor	National
16	Hungary	Policy advisor	Municipal
17	Hungary	Knowledge coordinator	National
18	Italy	Policymaker	National
19	Italy	Policymaker	National
20	Italy	Policy advisor	National
21	Italy	Policy advisor	National
22	Italy	Policy advisor	National
23	Macroregional	Policymaker	Macroregional
24	Netherlands	Policy advisor	National
25	Netherlands	Policy advisor	National
26	Netherlands	Policy advisor	National
27	Netherlands	Knowledge coordinator	National
28	Netherlands	Knowledge coordinator	Municipal

* We divided the interview partners into three main categories: (1) policy-makers determining policies and practices at supranational, national or sub-national level in the field of innovation and digitalisation; (2) policy advisors informing policy-makers at supranational, national or sub-national level on various issues involved in their relevant policy field of innovation and digitalisation; (3) knowledge coordinators acquiring and transferring relevant scientific information to policy-makers and analysts working in the field of innovation and digitalisation at supranational, national or sub-national level, after assessing the data integrity and reliability.

The indicator ‘social trust’ (based on the country-level valuation of the ESS question ‘would you say that most people can be trusted, or that you can’t be too careful in dealing with people?’) was chosen due to the perceived importance of generalized trust in improving the scientific evidence-to-policy divide. The level of generalized trust in society also indirectly influences how policymakers tend to believe in or engage with certain sources of (scientific) information, shaping the institutional settings and personal circumstances around evidence-informed policymaking (see also Introduction). Gathering data by primary and secondary research in four countries featuring variations in the level of generalized social trust aimed to enhance the generalizability of the resulting trends, with the international interviews enhancing the reliability of our findings by providing a lookout to international trends.

In addition to country-level variety, the study aimed to involve a broad and balanced mix of interviewees from each country, engaging both policymakers and policy-influencers in innovation policy and digitalisation, specifically in three topics close to the main objectives of the TRESCA project, namely digital safety, environ-mental health, and the future of skills and work. The identification of national interview partners was the prime responsibility of the relevant partners, i.e., Centre for Social Innovation (ZSI) in Austria and Hungary, Observa in Italy, and Erasmus University Rotterdam in the Netherlands, with the three organisations making a final common decision on the preferred interview partners. The main inclusion criteria were that the interview partner should be active in one of the above-mentioned fields, should ideally possess relevant experience (reached at least a mid-term position or has been working with a given topic for more than five years) and be either in a direct decision-making role or act in a close advisory or knowledge coordinator position to policymakers at national or sub-national level. No specific exclusion criteria were approved. The national interview partners were contacted by each of the responsible partners through e-mail or phone.

Based on these inclusion criteria, the interview partners working at an international level were primarily recommended by the European Commission’s responsible project officer and ultimately chosen and interviewed by ZSI. The international-level interview partners were contacted by ZSI through e-mail or phone.

Altogether 28 interviews were conducted between May and July 2020 with the following country breakdown: seven international, six Austrian, five Dutch, five Hungarian, and five Italian (due to the ongoing COVID-19 pandemic, all interviews were conducted online using various platforms such as GoToMeeting, Microsoft Teams, Skype, and Zoom, depending on the interviewing partner organisation’s licenses and the interviewee’s preferences; the interviews generally lasted around one hour).

The interviewees were asked about how they consume, frame, and use information stemming from various science communication channels and produced in a variety of formats, and on whether this information helped them define their policy options and take relevant decisions. The science communication consumption patterns of interviewees were considered, accompanied by their perceptions and engagement with scientists and science communication channels.

The interview data were analysed using qualitative content analysis. After asking general background questions from the interviewee, the interviews focused on the following six main question categories: (1) the most relevant science communication data sources and data collection processes used for decision-making, (2) the most relevant scientific topics with regard to interviewee’s work, (3) the most relevant science communication channels and formats used for decision-making, (4) the most effective engagement methods between science and policy as perceived by the interviewee, (5) strategies and policies applied to communicate policy decisions to scientists and potentially journalists, (6) the relevance of open science or RRI principles as perceived by the interviewee.

The interviews were recorded by handwritten transcripts or otherwise, and – following a pre-approved uniform interview guide – the raw data was summarized in English in two steps: first, a maximum two -three-page long summary was compiled by each partner, focusing on the most important take-aways of each question category mentioned above ( Szüdi *et al*., 2022b). Second, the most important findings of each interview were summarized around the following key categories: data sources, policy development process, stakeholder involvement, trust in science and science communication, strategies and approaches in communication with journalists, key elements, key challenges, and opportunities of science communication in relation to policy. The key findings were then checked and cross-referenced between countries to generate more granular categories within the key trends. At the end of the summary process, the most relevant quotations were translated to English to showcase the main trends in the science-policy nexus, which formed another chapter of the same report ( Szüdi *et al.*, 2020).

As detailed below in the Results section, three main trends were identified, namely a more institutionalised and stronger engagement between the policy and scientific actors, a shift towards scientists engaging in more open, reliable and accountable science communication practices with policymakers, and the enhanced digitalisation and visualisation of science communication with policymakers.

With the help of the identified trends detailed along the above categories, conclusions were also drawn for the policy level. These conclusions were summarized in an initial policy brief in February 2021, also including short but concise policy recommendations on how to better engage with scientists and communicate scientific findings that can inform key policy decisions in the field of innovation and digitalisation. The initial policy brief was updated and finetuned in February 2022 after a European-level consultation process with policy-maker stakeholders, involving online presentations and other dissemination activities, prominently including an online feedback option to which 37 experts responded from 18 countries (Austria, Belgium, Bosnia and Herzegovina, Denmark, Germany, Greece, Hungary, Italy, Montenegro, Netherlands, Serbia, Slovakia, Slovenia, Spain, Sweden, Ukraine, United Kingdom, United States) and the European Union (EU), resulting in a finalised version of the policy brief ( Szüdi *et al.,* 2022a).

We must underline the research limitation that – even though the science-policy nexus is rapidly evolving in each corner of the world – the focus of this article is on more developed countries that make up most of the members of the international organisations analysed in the secondary research phase (e.g., EU bodies or the Organisation for Economic Co-operation and Development (OECD)) and are present in the interview and consultation phases. Thus, the below described science communication trends and their relations to policymakers, as well as the policy recommendations are mainly valid for developed countries.

Another limitation stems from the fact that the interviews were taken with policymakers engaged in the fields related to innovation policy and digitalization. Therefore, the science communication trends and the policy recommendations should be interpreted in the framework of these scientific areas that might not be readily generalizable to other fields.

The final limitation had arisen because the COVID-19 pandemic made the conduction of face-to-face interviews near impossible at the time of our research. Hence, online interviews were held with the interview guides specifically prepared for carrying out and analysing online semi-structured interviews.

## Results

Based on the data collected through our primary research consisting of 28 semi-structured interviews with relevant policy-makers and policy influencers from four countries and at an international level ( Szüdi *et al*., 2022b), as well as thorough secondary research analysing 109 academic and non-academic publications, we identified the ongoing and emerging communication trends between scientists and policymakers in the field of innovation policy and digitalisation in the following main categories:

### Stronger engagement between science and policy

Most scientific advice is provided to policymakers through ‘scientific assessment’, i.e., an expert assessment of the state-of-the-art of knowledge in a given field, as well as the implications of such knowledge. There are many ways to communicate these scientific assessments to decision-makers. Scientific advice is provided through a range of different mechanisms, dependent on institutional, political and cultural factors ( Allio *et al.,* 2006).

While fully acknowledging the view of Trench (2008) that more science communication models can simultaneously co-exist in various institutional settings we maintain that there is a movement towards including scientific evidence in the legislative and regulatory policy-making process by institutionalising the scientific advisory function within policy-making bodies. A stronger engagement between scientists and decision-makers is built through various institutional mechanisms to ensure the integrity, quality and effectiveness of scientific communication systems.

These institutional mechanisms are built on dialogue and engagement that complements and replaces the traditional communication models based on ‘deficit’ theory. The ‘deficit’ theory in the context of the science-policy communication argues that the lack of available scientific information hinders policymakers to consider scientific data more prominently in their decisions – the mitigation of such a gap is sufficient to increase the uptake of scientific evidence in policy decisions ( Reincke *et al.,* 2020). Modern science communication theorists heavily criticize this approach and claim that the use of scientific information in policy-making does not only depend on the available information, but also involves coalition-building, rhetoric and persuasion, accommodation of conflicting values and expectations ( Contandriopoulos *et al.,* 2010) – a perspective that is also in line with the theoretical framework around ‘trust, translation, and timing’ by Hinrichs-Krapels *et al.* (2020) that informs our perspective.

Institutional mechanisms facilitating a stronger engagement of scientists and decision-makers can be categorized into two main forms:

(1) brokering where certain persons or boundary organisations bridge science and policymaking by providing information and developing relationships between knowledge producers (risk assessors) and knowledge users (risk managers) while staying independent from the interests of both sides. Boundary organisations such as non-profits, industry groups, advocacy organisations, journalists or media organisations facilitate the flow of information among scientists, policymakers, and other stakeholders while staying independent in relation to each stakeholder ( Bednarek *et al*., 2016).

The use of intermediary organisations varies in the interviewed countries, for instance in Hungary technology transfer offices (of universities) and consultants (selected through procurement procedures) were mentioned as bodies taking on science communication tasks with decision-makers in R&I, while an Austrian interviewee (no. 2) from a national-level funding organisation for basic research highlighted the benefits of close cooperation with journalists, which is *“*an effective instrument to create impact, in part because you are then harder to be ignored by other decision-makers” ( Szüdi *et al*., 2022b).

Other interviewees mostly agreed with the function of journalists in creating impact but their specific importance was judged differently.

More interviewees were of the opinion that such brokering is beneficial and required to circumvent the still existing mistrust (or misunderstanding) between scientists and policymakers. From the policy perspective the issue seems to be with scientists respecting the ‘hierarchical order’ too much and not engaging in direct communication with policymakers even though it would serve their interests. They tend to rely on the official channels of their (scientific) institution to do the necessary communication with policymakers. Boundary organisations or guidelines supporting translation (see next trend) could remedy this problem.

Regarding concrete examples at the EU level, the Joint Research Centre (JRC) represents the first boundary organisation which – as the EU’s in-house research service – has had a formal science advisory role since 1988. JRC is at the interface of science and policy giving scientific advice to more than 20 Directorate Generals (DGs) without having an own political agenda. JRC’s activities cover policy anticipation, policy formulation, policy implementation and ex-post policy evaluation within a range of EU policies. JRC also has its dedicated skills and training agenda, the Knowledge Management for Policy (KMP) programme to identify ways for boundary organisations to connect the supply and demand side of policy-relevant knowledge ( Topp *et al*., 2018).

The European Parliament’s 27-member Panel for the Future of Science and Technology (STOA) is another EU-level boundary organisation which – with the support of external experts – focuses on providing the EP with high-quality independent studies and identifying options for the best courses of action since 1987. Political oversight is ensured by the EP which decides on STOA’s research priorities and approves its studies ( Wilsdon & Doubleday, 2015).

Another wave of establishing institutions for brokering started in the mid-1990s when independent scientific committees were set up to provide scientific advice for the preparation of policy and regulatory advice required by legislation. At the same time scientific advisory agencies were set up, such as the European Medicines Agency and the European Chemicals Agency and internal advisory bodies, such as the European Group on Ethics of Science and New Technologies ( Rogers, 2011). The novelty of these agencies was the delegation of executive power to carry out risk assessments to a neutral agency while the responsibility for policymaking (risk management) remained at the Commission.

Our interview partners at the EU level all confirmed that they do not have a formal list of data sources for scientific input and communication but heavily rely on data sources deemed the most trustworthy such as the above-mentioned boundary organisations, as well as other bodies such as Research Executive Agencies.

(2) partnership building where more permanent relationships among scientists, policymakers, and practitioners are built that can directly benefit science communication by raising understanding of science and trust: scientists come to better understand local needs and circumstances, while policymakers gain a better understanding of the process of research. This increased trust and better understanding make it possible to design research agendas and protocols responsive to the needs and goals of all parties ( National Academies of Sciences, 2017).

The concrete forms of such partnerships vary per country as became apparent in our interviews. For instance, in Austria the research platforms as official networks bring together policy and scientific stakeholders in the policy-making process, and there is an ongoing attempt in Hungary to build up territorial platforms around local innovation centres (usually universities or/and research centres) comprised of all actors along the quadruple helix (public bodies, municipalities, start-ups, businesses, scientists).

At the EU level, based on the success of JRC and following the recommendations of the Commission Working Group “Democratizing Expertise and Establishing Scientific Reference Systems” a Scientific Advice Mechanism (SAM) was established in 2015. The aim was to move forward from the brokering role of JRC towards a real partnership between science and policy at the EU level, and the design of SAM took into account the shortcomings of its short-lived predecessor, the Chief Scientific Adviser function.

SAM consists of seven high-level experts whose work is supported by a 15-person Secretariat. SAM is fully embedded within the Commission’s system: it is located at DG Research and Innovation and has close connections to the College of Commissioners (direct reporting and operational support), as well as a structured relationship with national academies (SAPEA) and other Member State bodies ( Klumpers, 2015).

SAM represents a close partnership between scientific opinion and high-level policymaking. Scientific advice is communicated in various formats, proactively choosing its agenda, and advising the Commission whereas the responsible Commissioner acts as an intermediary between supply and demand of scientific input.

During one of our interviews (international interviewee no. 1, serving in mid-level management position in more European public bodies dealing with innovation), the example of the European Institute of Innovation and Technology (EIT) came up which brings together businesses, research institutes and higher education to foster Europe’s innovative edge. The EIT predominantly uses scientific information coming from its own paying member organisations, which in itself shows a really strongly internalized science-policy process for evidence-informed policymaking in various fields related to innovation.

### More open, reliable and accountable science communication practices with policymakers

The stronger engagement of scientists with policymaking via more institutionalised arrangements may be understood as a strategic alliance-building process with experts to enhance the competence and legitimacy of a given organisation ( Moodie, 2016).

This trend entails two main risks: on the one hand, as civil society and trade union groups point out, corporate interests influencing expert groups may increase in relevance at the national and EU level ( Moodie, 2016). At the same time, there is a risk of technocratic bias when broader societal implications and values are not taken into account by policy decisions based on scientific evidence.

The former risk for increasing lobbying power was more frequently mentioned in interview countries where personal relationships seem to have a higher role in evidence-informed policy-making (i.e. Italy and Hungary), but Dutch interviewee no. 3 (a national-level senior policy officer) also pointed out that lobbying organisations might often pose challenges in using relevant scientific evidence when they consider this contrary to their strategies and interests: “The ones that actively lobby are often the problems in the field - they feel that the policy/law will obstruct them and they are trying to convince you to change the policy you are developing.” ( Szüdi *et al*., 2022b).

It seems so that in all analysed countries conflicts of interest may occur when policy-making conflicts with the strategies and actions of the organisations represented by lobbies, which decreases the procedural and outcome effectiveness of evidence-informed policymaking.

To address such risks, there is a need for a science communication in the policy sphere, which is in itself open, reliable and accountable (process dimension) and is based on open, reliable and accountable data (input dimension). This should ensure that evidence-informed policymaking does not fall victim to specific lobby interests, and scientists and policymakers can cooperate on equal footing with open data and processes although the risk of so-called hidden agendas is always possible ( McConnell, 2018).

Concerning the process dimension *,* there are ongoing attempts to increase the openness of evidence-informed policy-making processes by making the related communication processes more accountable. Openness was also increased by engaging a broader spectrum of stakeholders, such as scientists, decision-makers and knowledge transfer specialists into the formulation of recommendations.

Our interview findings confirm that the form and intensity of engagement has been increasing in recent years, shifting from mere consultation or engagement through traditional formats, such as expert groups, panels, boards, committees or meetings to more co-creative interactive formats, including the use of scientific ambassadors by the European Parliament, the organisation of the #EUvsVirus Hackathon by the European Commission, or the conducting of a series of roadshows in Hungary where decision-makers for innovation policies directly meet with local academic and scientific communities. Due to the COVID-19 pandemic, the previously rather negative opinions on online meetings have also changed and more and more meetings between scientists and policymakers take place online for various reasons such as higher interactivity, the use of more visual solutions, time efficiency, cost benefits or a higher level of flexibility.

Austrian interviewee no. 3 (a high-level member of the research policy advisory board) explicitly referred to 2008 as a cut-off date for such a paradigm shift towards more co-creative approaches in the science-policy nexus when stating, “[…] before 2008, the ministries developed the political measures on their own and presented them afterwards. Nowadays, it is commonly agreed that researchers [and citizens] have to be engaged as early as possible to gain more creative insights and solutions.” ( Szüdi *et al*., 2022b).

With the aim of fostering a more open and accountable evidence-informed policymaking, a series of EU-level guidelines were published with practical recommendations on the effective presentation of scientific advice to policymakers ( EC, 2004; EC, 2008; EC, 2010). Such guidelines conceptualized the key priorities for deepening communication and strengthening the transfer of knowledge and experience between scientists and policymakers. They mostly highlighted practical means to use within projects in the field of research and innovation but did not provide a deeper understanding of how policy-making systems and processes function. A more comprehensive, easy-to-use guide covering both institutional and legislative structure and procedures, as well as practical communication tips and hints is still missing at EU level.

The mutual understanding of both parties involved in the evidence-informed policy-making process was also fostered by such initiatives as the MEP-Scientist Pairing Scheme started in 2009 by STOA. This scheme aims to support the development of relationships between members of the European Parliament (MEPs) and scientists by letting scientists shadow their MEPs during their parliamentary business. The shadowing exercise aims to improve access to scientific advice and deepen scientists’ understanding of the role of science in policymaking. Scientists can attend committee meetings and meet with officials working in their relevant policy areas and, *vice versa*, while MEPs visit the workplace of scientists to experience how research is conducted in practice.

To support openness and accountability in its evidence-informed policy-making process, the European Commission made its online register of experts and expert groups (organisations and its representatives) publicly available in 2005.

With regard to the input dimension, open science is gaining momentum, also transforming science communication with policymakers, giving rise to the practice of open scholarly communication addressing not only access to data, but also scholarly outreach and engagement with decision-makers ( EC, 2016). The European Commission has recently introduced the European Open Science Cloud (EOSC) and the Open Research Europe (ORE) publishing platform for ensuring an open, reliable, virtual, federated environment to store, share and re-use research data across borders and scientific disciplines and provide access to a rich array of related services with new opportunities arising in the science-policy interface.

The COVID-19 pandemic gave further impetus to open science trends in evidence-informed policymaking at the EU level. For instance, the European Commission and EMBL’s European Bioinformatics Institute (EMBL-EBI), together with EU Member States and research partners started their own dedicated data portal facilitating open data sharing and analysis concerning coronavirus research ( https://www.covid19dataportal.org/).

The use of open-source platforms and an open data portal in evidence-informed policymaking was underlined by several Austrian interviewees (working both at national and regional level) who deemed these an increasingly important data source. Hungarian policymakers also repeatedly mentioned the increasing relevance of open-access sources and methods.

The COVID-19 pandemic put further pressure on scientists to inform policymakers on all new related scientific findings as fast as possible – the relevance of timing is growing. Italian interviewee no. 2 (a member of the National Research Council) bluntly put it, “the newsworthiness of a scientific fact is not associated with the timing of the scientific community” ( Szüdi *et al*., 2022b).

This interviewee shows how information provision has had to speed up, resulting in turn in an even more rapid diffusion of science-related data and information in open access publications or social media outlets (for the latter see also next trend). Open-access publication venues entail the risk of less rigid pre-publication review and verification of facts than in the case of traditional venues such as peer-reviewed journals. Even the biggest names in the business fall victim to the pressure of publishing relevant scientific results first, as showcased by a retracted article on hydroxychloroquine in Lancet in June 2020.

Fact-checking gains in relevance in parallel with more open science communication practices or the spread of social media channels. One interviewee in Austria (Austrian interview no. 4, senior manager in a public funding organisation of research and innovation) explicitly raised the issue of elaborating a strategy for ensuring the credibility of open access and social media sources. ( Szüdi *et al*., 2022b). The most relevant new EU initiative in this regard is the European Digital Media Observatory (EDMO), which started its operation on 1 June 2020. The EDMO brings together fact-checkers, academic experts, media organisations and other relevant organisations to provide support to policymakers. It has been set up in the framework of the 2018 EU Action Plan on Disinformation. The EDMO promotes scientific knowledge on online disinformation, advances the development of fact-checking services and supports media literacy programmes.

Another initiative strengthening a more open and accountable evidence-informed policy-making process is the EUvsDisinfo platform – the flagship project of the European External Action Service (EEAS) – raising public awareness and understanding about disinformation, including scientific information. The expert team behind the website is also engaged with policy outreach. The experts brief and train EU institutions, Member State governments and other policy actors, infusing accountability and reliability to the evidence-informed policymaking at national and EU level.

### The increasing digitalisation and visualisation of science communication

As already mentioned in the Introduction section, the proper presentation of reliable facts is not in itself enough for a successful communication with policymakers. Translation in a sense means that scientific evidence should be repackaged in a digestible and accessible format, providing a narrative to policymakers to which they can relate.

As one Dutch interviewee (Dutch interview no. 1, a policymaker at a national ministry) stated *,* “what becomes more important with quantitative data is building a narrative. Key figures do not tell the complete story, so it is important to find a narrative. Systematic collection of good and bad examples is therefore imperative – and to combine with the key numbers into a narrative” ( Szüdi *et al*., 2022b).

In short, narratives are especially relevant to quantitative results, according to this interviewee, to provide the appropriate context to numbers that might be difficult to interpret for policymakers or other stakeholders.

The important role of narratives in communicating science with non-expert audiences has been underlined by several scientists in recent years ( Brounéus *et al.*, 2019; Dahlstrom, 2014; Davies *et al.*, 2019), and several interrelated factors, such as the advent of big data and the related data visualisation techniques, and the changing roles and landscape of boundary organisations made the narrative-building more widespread. Visual and interactive solutions present complex data in transparent and eye-catching formats through novel digital channels.

As one interviewee at the international level (European interview no. 1, serving in mid-level management position in more European public bodies dealing with innovation) told us, “the background noise gets louder in the field of digitalisation, and in the entire STEM field, therefore there is a growing need to communicate in a clearer, simpler, more engaging way” ( Szüdi *et al*., 2022b).

The interviewee mentions that this demand for a simpler but more engaging science communication led to the growing role of visual solutions, as well as to other innovative formats often taught to scientists at leading academic organisations, such as elevator pitches (through which scientists are able to sell their ideas in an engaging way tailor-made to policy target groups).

Our interviews in general confirmed that even traditional and extensive reports with many long annexes and list of detailed graphs are increasingly replaced by shorter ‘agenda’ style documents with fewer details, represented by more visualisation. Such visual methods may include graphs, charts, process diagrams, maps, portraits or even videos.

This kind of communication between scientists and policy-makers – carried out through digital means and with the support of visual solutions – was fostered by big data and the related new data presentation opportunities. This ongoing trend was strengthened by the COVID-19 pandemic when timely and reliable data was needed in easily understandable formats. For instance, data visualisation enabled and necessitated by big data supported willing public organisations to quickly establish and regularly update new web platforms on COVID-19. The World Health Organisation (WHO) has its own COVID-19 dashboard where interested people can explore the latest information on the virus in various visual forms and graphs (see: https://covid19.who.int/). Due to the broad networking of WHO, policymakers are one of the primary target groups of such visualised data sets provided by scientists.

The changing role of boundary organisations also facilitates the shift towards visualized science communication. On the one hand, as part of our first main trend, the more institutionalized relationships between scientists and policymakers also involve partnerships between public organisations performing the data collection and other (often private) organisations more able and inclined to translate this (raw) data to modern visual formats. For instance, data collected by the scientists of the European Centre for Disease Prevention and Control (EU CDC), an independent agency of the European Union has been extensively used by Our World in Data, a scientific online publication which provides interactive charts and maps to present research findings in an open and non-profit way to its readers, among them stakeholders from the academic and policy spheres.

On the other hand, digitalisation makes it possible to decrease or eliminate the role of traditional boundary organisations such as journalists, the long-term effect of which is still too early to evaluate. Scientists are increasingly using social media, personal websites or blogs for direct communication of their findings. Social media platforms used by scientists might include ‘general’ channels such as Twitter but also platforms such as ResearchGate that are specifically aimed at academic people where they can network and share their latest research with other interested peers, including experts from the policy sphere.

One specific web platform for a broader science-public engagement – with specific policy implications (at least in the US) – is the Reddit Science community’s Ask Me Anything (AMA) series. Reddit is primarily a US entertainment social network and news website where registered users can vote posts up and down to determine their popularity and relevance. Reddit has specific topics, among which Science is quite popular with more than 19 million users. Since it has a system of verification, scientists tend to post reliable and accountable comments on Reddit in an open online environment. Thus ‘ordinary’ users can distinguish between verified expert opinion and random comments, with the support of voluntary moderators.

The AMA series are basically crowd-sourced interviews where users could ask experts any questions in their related scientific expertise areas (pre-screened by moderators with at least a bachelor’s degree in related science). The format and perceived image of reddit helped spread verified scientific information through the forums and the AMA series, which was well-received even by the most renowned scientists; for instance, Stephen Hawking took part in AMA. The AMA provides a channel where interested citizens, scientists, science communicators, business and policy stakeholders can engage in a broad field of scientific issues in an informal but moderated environment. In 2017, there was a dedicated AMA on the topic of empowering scientists and engineers to engage in policy. More than 300 comments were answered by two high-level experts engaged in the science-policy interface.

This shows that in cases where the public or the media catch up with some topics which go viral then policymakers may also have a hard time ignoring these scientific findings in their policymaking. *Vice versa*, policymakers may also decide to inform the public on scientific facts through their personal social media channels. If this information gets distorted through political or economic lobbying, group interests or value preferences, scientists may also have to fight hard to correct or protest against misinterpreted scientific findings ( Brossard & Lewenstein, 2009).

We have gathered mixed interview results when confirming the more prevalent use of social media by policymakers in gathering scientific findings: it is still undoubtedly true in all interviewed countries that traditional data sources are the most relevant (mainly due to their perceived reliability and supposed fact-checking undertaken by scientists – see trend 2), but several policymakers mentioned the use of non-traditional online sources such as social media. This was more likely in Austria and Hungary than in Italy and the Netherlands. However, Dutch interviewees interestingly mentioned the increasing role of social media in another context, namely by influencing the agenda and urgency of certain innovation topics taken up by policymakers. As one Dutch policymaker (Dutch interview no. 1, a policymaker at a national ministry) said, *“*some topics would not have entered the public sphere at all if social media had not been this widely adopted” ( Szüdi *et al*., 2022b).

### Policy recommendations

Based on the above-detailed primary and secondary research results, specific policy recommendations aimed at the decision-makers responsible for innovation policy and digitalisation at national and EU level were compiled. Our aim was to contribute to a more effective and efficient uptake of scientific findings in evidence-informed policymaking by pointing out the main challenges and the potential (practical) solutions that can be initiated, strengthened, or continued by policy actors.

The relevance and usability of the proposed recommendations were tested through a (predominantly online) consultation process with policymakers who were invited to give their overall evaluation and detailed opinion on each proposal. The list below presents the policy recommendations deemed most relevant by the consulted policy actors: ^3^

- Create training opportunities and tailor-made learning resources for scientists and policymakers to learn each other’s vocabulary

One main obstacle in the translation of scientific evidence to valuable input used by policymakers in their policy-making process is the lack of understanding of the methods, terminology and related aspects of articulation by both sides in the science-policy nexus. By drawing on existing good practices, such course materials or other knowledge transfer initiatives – such as job shadowing exercises – should be developed that lay a specific focus on understanding the vocabulary and perspectives of both sides.

Self-reflection tools such as the Smart4Policy tool developed under the leadership of JRC are practical online questionnaires that enable both policymakers and researchers to understand their own competences in various fields required for evidence-informed policymaking. They lay down a competence framework and assessment method which might be useful for developing courses with the aim of mitigating the gap of understanding between science and policy.

To foster a truly open and accountable science communication in the policy sphere, such courses should be taken up from the earliest time possible, e.g., they could be included in Master or PhD programmes or offered for early-stage scientists, and they should not avoid complex topics, such as the involvement and communication of broader concerns, stakes and uncertainties of a scientific field to the relevant decision-makers. The TRESCA project has also developed its own MOOC (massive online open course) “Communicating Trustworthy Information in the Digital World” where one module specifically deals with science communication with policymakers. ^4^

- Leverage the use of digital media to create easily digestible and accessible, visualized science communication content

The emergence of big data and the related data visualisation techniques, coupled with the new opportunities provided by digital media make the translation of scientific findings to policymakers faster and more comprehensible. In case the perspectives and needs of policymakers are well understood by involved scientists – where training opportunities and guidelines can be of help (see relevant recommendation) – the most important take-aways can be presented in a multitude of formats, such as infographics, process diagrams or maps.

In line with the stronger engagement between scientists and policymakers, new technological solutions can be also used to support two-way dialogue and engagement by creating virtual meeting spaces, such as webinars, virtual cafés, online consultations or more informal online options, depending on the depth and frequency of the collaboration (brokering or partnership).

- Prepare short but comprehensive science communication guidelines aimed towards both scientists and policymakers

Such guidelines would not just ease the translation process but would also enhance trust in the science-policy nexus and foster a more open and accountable evidence-informed policymaking. We recognize the relevance of guidelines already elaborated on this topic at the national and EU level but advise to go one step further and not only describe practical advice and means to enhance the knowledge transfer through science communication but also provide a better overview for scientists on how policy-making systems and processes operate.

Lessons may be learnt from other parts of the world – for instance, the American Association for the Advancement of Science (AAAS) has already compiled a guide introducing the legislative process and bodies in the US Congress for scientists, followed by practical communication advices structured around ten goals ( White & Carney, 2011) – and professional science communicators can also support scientists’ and policy-makers’ better understand the institutional framework within which science communication functions.

- Strengthen the EC’s open science policy by encouraging open science activities of early-stage scientists

The ongoing shift towards more open science is also transforming science communication, giving rise to open scholarly communication addressing not only data access but also scientists’ engagement with policymakers. We acknowledge the huge steps made by the Commission towards open science (also in the framework of responsible research and innovation policies and practices) and would welcome the broadening of the scope of open science actions.

Within the framework of open science, the benefits offered by novel tools such as the European Open Science Cloud (EOSC) and the Open Research Europe (ORE) publishing platform should be promoted amongst early-stage scientists. Greater openness should also allow early-stage scientists to communicate their scientific findings when these diverge from the mainstream views, have unconfirmed hypotheses or contain many uncertainties.

- Promote the use of fact-checking websites and tools, in particular for controversial scientific topics

The enhanced openness in both the process and input aspects of science communication with policymakers bear the risk of diminishing the reliability and excellence of scientific input used for policy advice, in particular for the most controversial scientific topics such as the COVID-19 pandemic, migration or climate change. Fact-checking plays an essential role in addressing reliability issues threatening trust-building between the relevant actors.

The set-up and maintenance of such online platforms is also one of the main recommendations of the EU’s Action Plan against Disinformation. The use of such platforms should be a part of training and awareness-raising actions against misinformation and ‘fake news’. The newly established European Digital Media Observatory (EDMO) aims to facilitate the creation of networks of new fact-checking hubs to support the collaboration between academics, policymakers and media researchers engaged in evidence-informed policymaking.

- Promote new ways to motivate (early-stage) scientists to participate in science communication with policymakers

TRESCA research showed that the biggest obstacles for scientists to engage in communication with policymakers is lack of time, lack of training (see the recommendation on training opportunities), and the lacking or insufficient incentives (see: TRESCA report on the overview of (dis)incentives for scientists to engage in science communication) ^5^. The current academic incentive and reward system offers limited recognition to scientists for their science communication activities. Facilitating the already ongoing shift from traditional methods of measuring academic impact to new forms of alternative metrics may help in achieving better incentivisation.

Policymakers at national and EU level could support this paradigm shift by setting up dedicated schemes such as awards, prizes or grants to science communication activities, which can be considered a merit to be added to scientists’ CV. Such schemes should specifically target early-stage scientists in order to ignite the change process as early as possible.

## Discussion

We set out to understand how emerging science communication trends in the science-policy nexus influence the use of scientific findings in policy-making processes in the fields of digitalisation and innovation. Our findings show that not only communication scholars and professional science communicators discredit the deficit theory but in recent decades policy-making bodies and institutions have also embraced new methods of collecting and using scientific input in evidence-informed policy-making processes.

In line with the arguments advocating for a deeper mutual understanding of the operating conditions, needs and perspectives of both policymakers and scientists, such as the biased assimilation theory, such methods place great emphasis on building trust between the two sides involved. The enhanced trust will facilitate a more efficient translation of scientific findings to policy input and the assurance of a timely provision of robust evidence.

The common feature of such methods is that they do not want to solve the issue of underused or misused scientific data in policy decisions by providing more data but by collecting and providing data differently to policymakers. In short, scientific input is provided to policymakers differently than before: data is collected from a higher number of more open and reliable sources with the engagement of more actors and is provided through more permanent (institutionalized) relationships, in shortened and more visual formats, utilizing the opportunities provided by digital solutions.

This signals a general change from a linear communication model to a more institutionalized and systematic dialogue between scientists and policymakers which is embedded in local organisational relations, indicating that there is no ‘one-size-fits-all’ solutions and each country may have many and differing forms of science communication with policymakers.

Notwithstanding this complexity, we argue that these methods fall into at least three ongoing science communication trends, namely 1) a stronger engagement between scientific and policy actors (establishing or strengthening brokering or partnership arrangements), 2) the proliferation of more open, reliable and accountable science communication practices with policy-makers (covering both a more open and accountable scientific advisory process with an earlier and more accentuated engagement of a multitude of actors and the use of more open and reliable data sources), and 3) an enhanced use of digital and visual solutions in science communication with policy-makers.

One of biggest challenges threatening the sustainability and efficiency of these trends towards a sustained dialogue between scientists and policymakers is information overload. Information is becoming available in increasing volumes and at an increasing speed. In order to deal with such an increased flow of information, scientific input should ‘stand out’ and grab the attention of policymakers. This can only be achieved if scientists are motivated and well-trained for communication with policymakers.

There could be various bottom-up and top-down ways to encourage scientists to carry out outreach science communication with policymakers, such as the introduction of new alternative metrics or other incentives recognizing science communication efforts in career progress. The alternative metrics should be complemented with alternative ways of publishing scientific findings: the increasing number of open-access platforms – such as those established by the EU – offer such possibilities.

Once motivated to take part in science communication with policymakers, scientists should be assisted to perform communication as successfully as possible. Supporting structures may include practical tools such as guidelines that explain how to communicate compelling arguments in a clear (and potentially visualized) way to policymakers, or the provision of training and learning opportunities offering knowledge to facilitate the knowledge transfer process.

The enhanced amount and speed of available scientific data also raises questions of reliability. In order to ensure the reliability of data and to counteract misinformation, fact-checking platforms such as the European Digital Media Observatory (EDMO), as well as a more systemic and less fragmented data collection and analysis process is required.

However, the challenges related to information oversaturation also brings new opportunities to maintain and strengthen the positive aspects of the evolving evidence-informed policy-making process. The threat posed by misinformation and the value of reliable and open scientific data became more accentuated during the COVID-19 pandemic and might lead to beneficial changes where openness and reliability in evidence-informed policymaking becomes even more important.

The introduction and spread of more creative and open formats of stakeholder engagement may pave the way for a more strategic science communication between experts and policymakers where databases are readily accessible for research purposes in a way that is institutionally sound and ensures the protection of sensitive data. This change would help to foster the already ongoing paradigm shift towards more co-creation for evidence-influenced policy decisions in innovation and digitalisation policy.

It is too early to make a final judgement on the durability of this process. Policymakers nevertheless should ensure the engagement and participation of a wide range of scientific stakeholders, while keeping in mind to ensure that the collection, processing and sharing of personal data serves the public interest and is consistent with societal values. We provided our short list of recommendations in this article with the hope that it may enhance the durability of this process towards more co-creation in evidence-informed policymaking.

## Data Availability

Based on the approved consent form, taking into account the ethical approval given by the Ethics Review Committee of the Erasmus School of History, Culture, and Communication, the interview transcripts cannot be publicly shared due to the obligation to protect personal data. Since the potential group of interviewees was deemed to be quite narrow in the participating countries therefore data storing of interviews was only allowed in a summarized and anonymised form. The sharing of full transcripts could have jeopardized the confidentiality and privacy of interviewees by potentially exposing their identity. The anonymised interview summaries are available at in the data availability statement. Interested readers and reviewers may ask for more detailed but still anonymised interview summaries including coded background questions by writing to the corresponding author ( szudi@zsi.at). The pre-requisite of such data sharing is the signing of a non-disclosure form. Zenodo. TRESCA D1.2 Interview Data. https://doi.org/10.5281/zenodo.6555023 ( Szüdi *et al.,* 2022b). This project contains the following underlying data: TRESCA interview AUTO1.pdf (Interview with Austrian participant 1). TRESCA interview AUT02.pdf (Interview summary with Austrian participant 2). TRESCA interview AUT03.pdf (Interview summary with Austrian participant 3). TRESCA interview AUT04.pdf (Interview summary with Austrian participant 4). TRESCA interview AUT05.pdf (Interview summary with Austrian participant 5). TRESCA interview AUT06.pdf (Interview summary with Austrian participant 6). TRESCA interview EU01.pdf (Interview summary with European international participant 1). TRESCA interview EU02.pdf (Interview summary with European international participant 2). TRESCA interview EU03.pdf (Interview summary with European international participant 3). TRESCA interview EU04.pdf (Interview summary with European international participant 4). TRESCA interview EU05.pdf (Interview summary with European international participant 5). TRESCA interview EU06.pdf (Interview summary with European international participant 6). TRESCA interview EU07.pdf (Interview summary with European international participant 7). TRESCA interview HU01.pdf (Interview summary with Hungarian participant 1). TRESCA interview HU02.pdf (Interview summary with Hungarian participant 2). TRESCA interview HU03.pdf (Interview summary with Hungarian participant 3). TRESCA interview HU04.pdf (Interview summary with Hungarian participant 4). TRESCA interview HU05.pdf (Interview summary with Hungarian participant 5). TRESCA interview NL01.pdf (Interview summary with Dutch participant 1). TRESCA interview NL02.pdf (Interview summary with Dutch participant 2). TRESCA interview NL03.pdf (Interview summary with Dutch participant 3). TRESCA interview NL04.pdf (Interview summary with Dutch participant 4). TRESCA interview NL05.pdf (Interview summary with Dutch participant 5). TRESCA interview IT01.pdf (Interview summary with Italian participant 1). TRESCA interview IT02.pdf (Interview summary with Italian participant 2). TRESCA interview IT03.pdf (Interview summary with Italian participant 3). TRESCA interview IT04.pdf (Interview summary with Italian participant 4). TRESCA interview IT05.pdf (Interview summary with Italian participant 5). Zenodo. TRESCA D1.2 Interview Data. https://doi.org/10.5281/zenodo.6555023 ( Szüdi *et al.,* 2022b). This project contains the following extended data: TRESCA Interview Template.pdf (interview guidelines and questions used in this study). TRESCA Consent form template.pdf (Blank cope of consent form used in this study). Data are available under the terms of the Creative Commons Zero "No rights reserved" data waiver (CC0 1.0 Public domain dedication). Zenodo. TRESCA D1.2 Desk Research Documents (Reference list). https://doi.org/10.5281/zenodo.6596207 ( Szüdi *et al.,* 2022c). This project contains the following extended data: TRESCA D1.2_desk-research-documents. (Collection of documents selected for further analysis during the secondary research stage (reference list in .rtf, .bib, and .rdf formats). Data are available under the terms of the Creative Commons Zero "No rights reserved" data waiver (CC0 1.0 Public domain dedication).

## Footnotes

^1 ^TRESCA project ( https://trescaproject.eu/) was financed under the programme H2020 SWAFS n. 872855 lead by University of Rotterdam in cooperation with CSIC (Spain), Observa (Italy), ZSI (Austria).

^2 ^Respondents from each country (at least 1,500 people in a representative sampling frame) had to give a score between 0 and 10 for the statement “most people can be trusted or you can’t be too careful” where the higher number indicated higher trust in people. Social trust is considered high above 6, medium-level between 6-5 and low below 5. Therefore, the Netherlands had a high level (mean value of 6.21), Austria had a medium level (mean value of 5.54), and Italy and Hungary (mean values of 4.79 and 4.75) had a low level of social trust in 2018.

^3 ^The policy recommendations can be checked in their whole context within the TRESCA Policy Brief available at https://trescaproject.eu/results/

^4 ^The TRESCA MOOC is available at https://www.coursera.org/learn/communicating-trustworthy-information-in-the-digital-world

^5 ^The report is available at https://trescaproject.eu/wp-content/uploads/2021/07/TRESCA_D1.5_SciCom-disincentives-overview-report.pdf
