# Supplementary material for: New trends in science communication fostering evidence-informed policymaking
Source: Open Res Eur. 2023 Oct 24;2:78. Originally published 2022 Jun 16. [Version 2] doi: 10.12688/openreseurope.14769.2 (PMC10618634; doi:10.12688/openreseurope.14769.2)
Supplement: Peer review history

# Reviewer report: Reviewer response for version 2

**DOI:** 10.21956/openreseurope.18102.r35800

**Published:** 2023-10-31

**Authors:** Laura Smillie

**Affiliations:** 1 Joint Research Center, European Commission, Brussels, Belgium

Thank you for taking the points made in the review into consideration.

If applicable, is the statistical analysis and its interpretation appropriate?

Not applicable

Is the study design appropriate and is the work technically sound?

Partly

Is the work clearly and accurately presented and does it engage with the current literature?

Yes

Are the conclusions drawn adequately supported by the results?

Yes

Are sufficient details of methods and analysis provided to allow replication by others?

Yes

Are all the source data and materials underlying the results available?

Yes

Reviewer Expertise:

Science-informed policymaking, risk communications, EU digital policies and democracy.

I confirm that I have read this submission and believe that I have an appropriate level of expertise to confirm that it is of an acceptable scientific standard.

# Reviewer report: Reviewer response for version 1

**DOI:** 10.21956/openreseurope.15953.r32448

**Published:** 2023-08-16

**Authors:** Laura Smillie

**Affiliations:** 1 Joint Research Center, European Commission, Brussels, Belgium

Dear Authors,

 Many thanks for the opportunity to review your work.

 The reason I ticked “partly” in response to “Is the study design appropriate and is the work technically sound?” stems from your plain language summary approach, which has led to a paper that sounds quite opinionated and lacking references for strong statements e.g. page 3, second column, top paragraph; page 4, first column, bottom paragraph; page 10, first column, third paragraph.

 Please note the typo on page 4 in reference to Oliver & Cairney. I would recommend that your argument would benefit from the inclusion of Topp *et al.,* 2018 ^1^.

 On page 9, I recommend rather than referring to neutral, you refer to independence in relation to the role of EU agencies.

 Page 13 - I think your policy recommendations would benefit from the inclusion of the JRC’s work on competence frameworks for policymakers and researchers: https://knowledge4policy.ec.europa.eu/projects-activities/competence-frameworks-policymakers-researchers_en

 Some reservation about the lack of a caveat justifying the overall general conclusions that stem from considerably narrower fields.

 I hope this is helpful.

If applicable, is the statistical analysis and its interpretation appropriate?

Not applicable

Is the study design appropriate and is the work technically sound?

Partly

Is the work clearly and accurately presented and does it engage with the current literature?

Yes

Are the conclusions drawn adequately supported by the results?

Yes

Are sufficient details of methods and analysis provided to allow replication by others?

Yes

Are all the source data and materials underlying the results available?

Yes

Reviewer Expertise:

Science-informed policymaking, risk communications, EU digital policies and democracy.

I confirm that I have read this submission and believe that I have an appropriate level of expertise to confirm that it is of an acceptable scientific standard, however I have significant reservations, as outlined above.

## References

[1] : Knowledge management for policy impact: the case of the European Commission’s Joint Research Centre. *Palgrave Communications* .2018;4(1) : 10.1057/s41599-018-0143-3 10.1057/s41599-018-0143-3

# Response

**Published:** 2023-10-16

**Authors:** Gábor Szüdi

**Affiliations:** Centre for Social Innovation, Austria

Thank you for your review and the useful comments. We have taken into account all your comments and the following changes have been made:

 - in order to 'tone down' the article and not give an impression of being opinionated, references have been added and/or the text has been revised for all the indicated paragraphs (page 3, second column, top paragraph; page 4, first column, bottom paragraph; page 10, first column, third paragraph)

 - typo has been corrected on page 4

 - Topp et al. has been included as a new source to give more theoretical framework for our arguments on trust-building as a necessary prerequisite for an efficient evidence-informed policy-making, as well as on the beneficial utilisation of boundary organisations

 - the wording of 'neutral' has been changed to 'independent' on page 9

 - the Smart4policy self-reflection tool has been highlighted among the policy recommendations where the need for new courses is discussed

 - a further limitation on the use of interviews with policy-makers from the fields of innovation policy and digitalisation has been added

# Reviewer report: Reviewer response for version 1

**DOI:** 10.21956/openreseurope.15953.r32440

**Published:** 2023-06-22

**Authors:** Chiara Cadeddu

**Affiliations:** 1 Department of Life Sciences and Public Health, Universita Cattolica del Sacro Cuore, Milan, Lombardy, Italy

Dear Authors, 

 Congratulations for your work. It defines in a very good way what are the main issues to be considered in the field of evidence-based policymaking and in the complex relationship between policy and science. 

 There is an urgent need after COVID-19 pandemic to allow a balanced and always connected link between these two actors, and this should be well understood at a general level in Europe and worldwide. 

 The issue of trust is then crucial and a sufficient level of it from the public can be achieved only in a win-win situation for the two, policymakers and scientists. 

 I have just a pair of comments to improve the article. 

- The first one is a better defined structure in shorter subparagraphs, especially in the results. This could facilitate the reading of a long article like yours and the citations in future works. 
- The second one is the inclusion of the issues related to the cultural peculiarities of a country, which could deeply affect the policy-science dialogue. There are mentions of it, but I think a general discussion is missing and could be improved.  

If applicable, is the statistical analysis and its interpretation appropriate?

Not applicable

Is the study design appropriate and is the work technically sound?

Yes

Is the work clearly and accurately presented and does it engage with the current literature?

Yes

Are the conclusions drawn adequately supported by the results?

Yes

Are sufficient details of methods and analysis provided to allow replication by others?

Yes

Are all the source data and materials underlying the results available?

Yes

Reviewer Expertise:

Public health, planetary health, vaccination, health policy and management

I confirm that I have read this submission and believe that I have an appropriate level of expertise to confirm that it is of an acceptable scientific standard.
